# Juglone Encapsulation in PLGA Nanoparticles Improves Solubility and Enhances Apoptosis in HeLa Cells

**DOI:** 10.1007/s12013-025-01691-9

**Published:** 2025-02-14

**Authors:** Duygu Elif Yilmaz, Busra Gumus, Hasan Demirci

**Affiliations:** 1 Department of Nephrology and Medical Intensive Care, Charité – Universitätsmedizin Berlin, Berlin, Germany; 2 Department of Molecular Biology and Genetics, Faculty of Arts and Sciences, Yildiz Technical University, Istanbul, Turkey; 3 Institute of Functional Anatomy, Charité – Universitätsmedizin Berlin, Berlin, Germany

**Keywords:** Juglone, PLGA, nanoparticle, HeLa, anticancer, apoptosis

## Abstract

The anticancer potential of juglone, a naphthoquinone derived from walnut trees, has been extensively studied; however, its hydrophobicity and toxicity obstruct its therapeutic applications. This study aimed to overcome these challenges by encapsulating juglone into poly (lactic-co-glycolic acid) (PLGA) nanoparticles and evaluating their antiproliferative and apoptotic effects on HeLa cells. Juglone nanoparticles (JNP) were obtained by single emulsion solvent evaporation method. Its key physicochemical properties, such as particle size, zeta potential, drug loading, release yield, and encapsulation efficiency values were calculated as 207.45 ± 1.67 nm, −24.12 ± 2.21 mV, 47.80, 66.90 and 90.12%, respectively. JNP’s antiproliferative effects were compared to those of free juglone on HeLa cells. The calculated IC_50_ values for free juglone and JNPs were 17.07 µM and 20.64 µM, respectively. Both formulations exhibited comparable dose-dependent antiproliferative effects across the tested concentrations. However, the nanoparticle-based delivery system demonstrated enhanced apoptotic activity, as evidenced by increased caspase-3 activation and greater suppression of BCL-2 levels relative to free juglone. These findings were further corroborated by TUNEL and immunocytochemical analyses, which confirmed the superior apoptotic induction by the nanosystem. Collectively, the results highlight the potential advantages of PLGA-based nanoparticle systems for the delivery of juglone, thereby improving its water solubility—a key limiting factor for its use—while minimizing its toxicity. These findings offer a promising approach for its application as an effective anticancer agent via nanoparticle-based delivery.

**Graphical Abstract:**

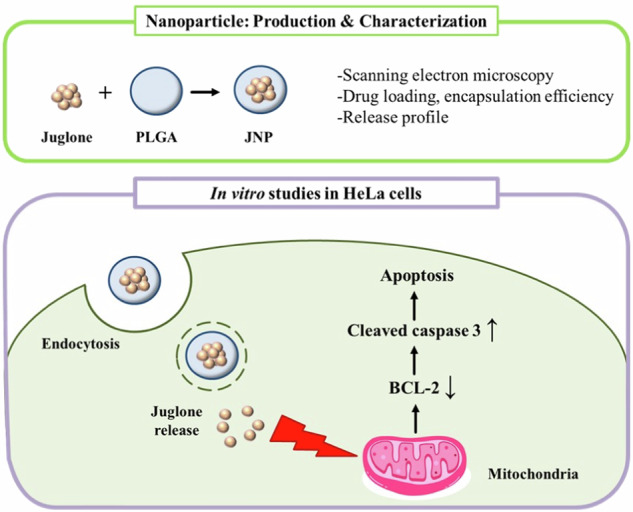

## Introduction

Cancer continues to be one of the most serious health challenges worldwide, with more than 19 million new cases and over 9 million deaths reported in 2020 alone [1]. Despite significant progress in cancer treatment, there is still a great need for new and effective therapeutic strategies for cancer.

Plants have long been a rich source of therapeutic compounds, and several plant-derived agents are widely-used in chemotherapy and cancer prevention [2]. Among these, the walnut tree (*Juglans* sp.) is particularly notable for its bioactive components, including quercetin, caffeic acid, naphthoquinones, and flavonoids [3]. One such compound, juglone (5-hydroxy-1,4-naphthoquinone), is a naturally occurring naphthoquinone with documented antimicrobial, antioxidant, antitumor, and anticancer activities. Juglone holds particular importance in cancer therapy due to its multifaceted mechanisms of action, including its ability to inhibit cancer cell proliferation, promote apoptosis, induce autophagy, suppress angiogenesis, and impede cancer cell migration and invasion across various cancer types, including pancreatic, gastric, cervical, and prostate cancers, as well as leukemia and melanoma [4–11]. Recent studies have shown that juglone can cause mitochondrial damage, leading to the release of cytochrome c (CytC). Once CytC enters the cytoplasm, it binds with Apaf-1 and procaspase-9 to form an apoptosome, which subsequently activates caspase-9 and caspase-3 [6]. Additionally, juglone has been demonstrated to suppress tumor angiogenesis and induce apoptosis in breast cancer cells through the regulation of the BCL-2/Bax signaling pathway [12]. Furthermore, juglone-induced apoptosis is triggered by the generation of the reactive oxygen species (ROS), leading to oxidative stress mediated by suppression of the PI3K/AKT pathway [13]. Moreover, juglone induces a disruption of the mitochondrial membrane potential, thereby activating of the intrinsic apoptotic pathway [5]. Previous studies have reported elevated levels of Pin1 (a prolyl isomerase) expression in several human cancers, including lung, breast, colon, and prostate malignancies. Notably, Pin1 is involved in activating various oncogenes and growth-promoting factors while simultaneously inactivating numerous tumor suppressors and growth inhibitors. Consequently, Pin1 ablation has been shown to inhibit cell growth and influence multiple cellular properties, such as drug sensitivity, motility, and metastatic potential [14]. The first Pin1 inhibitor discovered by low-throughput screening is juglone. Juglone functions to inhibit Pin1’s PPIase activity in the C-terminal catalytic domain, and a high dose of juglone reduces Pin1 protein expression [15]. Studies have shown that juglone can arrest the cell cycle at the G0/G1 phase, thereby inhibiting the proliferation of glioblastoma cells [16]. These promising properties position juglone as a compelling candidate for cancer therapy, particularly in addressing resistance to conventional pharmacotherapy. Its capacity to target numerous avenues and systems highlights its efficacy as a new anticancer drug.

However, juglone’s therapeutic potential is hindered by its hydrophobicity and associated toxicity, which limit its applications in biological systems [17]. Since juglone is an all-purpose molecule with broad implications for human health, research has been focused on overcoming these limitations. At this point, the use of nano-sized drug delivery systems offer promising results [18–20].

Biodegradable polymeric nanoparticles (NPs), such as those based on poly (lactic-co-glycolic acid) (PLGA), offer additional advantages, including biocompatibility, stability in circulation, and nonimmunogenic properties. They degrade into metabolites naturally processed by the body, reducing the risk of side effects [21–25]. Additionally, PLGA NPs are internalized by cells through multiple pathways, including fluid-phase pinocytosis and clathrin-mediated endocytosis, enabling efficient cellular uptake for the delivery of therapeutic agents into target cells [26, 27].

Recent studies have shown the potential of nanoformulations in addressing juglone’s limitations. Jahanban-Esfahlan et al. reported significantly lower cytotoxicity of juglone-loaded bovine serum albumin nanoparticles compared to free juglone in A431 and HT29 cells [28]. Similarly, our research group previously demonstrated that juglone-loaded nanosystems not only enhanced antifungal activity but also reduced toxicity by increasing the compound’s water solubility [29, 30]. Zhao et al. highlighted juglone’s ability to inhibit invasion and metastasis in HeLa cells, emphasizing the need for further exploration of its molecular mechanisms [9].

Building on these findings, the present study aimed to investigate the anticancer potential of juglone-loaded PLGA nanoparticles (JNP) in HeLa cells, a human papillomavirus (HPV)-positive cervical cancer model. In this study, we sought to evaluate the antiproliferative and apoptotic effects of juglone nanoparticles in detail, with the goal of advancing their development as a potential nanotherapeutic for cancer treatment.

## Materials and Methods

### Nanoparticle Production

Due to the strong hydrophobicity of the juglone molecule, the nanoparticles were produced by the single emulsion (w/o) solvent evaporation method as described in our previous publications [29, 31, 32]. For the production of juglone loaded nanoparticles, a mixture of 100 mg of PLGA (Sigma-Aldrich) was dissolved in 1 mL of dichloromethane (DCM; Sigma-Aldrich) and 55 mg juglone was mixed (Sigma-Aldrich) in 1.5 mL of DCM. This organic phase was added dropwise into 4 mL of 3% polyvinyl alcohol (PVA; Sigma-Aldrich) solution maintained in an ice bath with concurrent sonication at 80% power for 90 sec. The resulting oil-in-water (o/w) emulsion was then added drop by drop into 35 mL of 0.1% PVA solution on a magnetic stirrer. This mixture was incubated overnight, allowing the organic solvent to evaporate. The solution was subsequently centrifuged at 9000 rpm for 40 min, and this process was repeated three times, with the pellet washed with ultrapure water after each step to ensure purity. Finally, the pellet was lyophilized and stored at −20 °C for subsequent analysis.

### Nanoparticle Characterization

#### Encapsulation efficiency, reaction yield and drug loading

The encapsulation efficiency (EE), the reaction yield (RY), and the drug loading (DL) were calculated according to the formulas given below:

1 $${EE} \% =\frac{{Encapsulated\; juglone\; in\; NPs}({mg})}{{Initial\; juglone\; amount}({mg})}\,\times\,100$$

2 $${RY} \% =\frac{{Amount\; of\; nanoparticle\; produced}({mg})}{{Amount\; of\; initial\; polymer}+{juglone}({mg})}\,\times\,100$$

3 $${DL} \% =\frac{{Encapsulated\; juglone\; in\; NPs}({mg})}{{Amount\; of\; NPs\; produced}({mg})}\,\times\,100$$

#### Particle size, zeta potential, polydispersity, and SEM analysis

A Zetasizer (Zetasizer Nano ZS, Malvern, UK) was used to measure the zeta potential of the nanoparticles via the electrophoretic light scattering technique, while the dynamic light scattering technique was employed to determine the mean diameter. The shape and surface morphology of the nanoparticles were analyzed using scanning electron microscopy (SEM; JSM-7001FA, Jeol, Japan), as described in a previous study [33].

#### Release study

The drug release profile of the JNPs was determined using a modified dissolution method [29]. Briefly, 10 mg of JNPs were mixed with 4 mL of PBS (150 mM, pH 7.4) and incubated in a shaking incubator at 37 °C with constant agitation at 150 rpm. At designated time points (1^st^, 2^nd^, 3^rd^ hours, and 1^st^, 2^nd^, 3^rd^, 4^th^, 8^th^, 11^th^, 14^th^, 17^th^, 25^th^, 37^th^, 60^th^ days), the solution was centrifuged at 9000 rpm for 20 min. The absorbance of the supernatant was measured at 424 nm using a UV−Vis spectrophotometer to calculate the amount of juglone released.

#### Cell culture and treatment

HeLa cells (ATCC) were cultured in DMEM medium (PAN Biotech) supplemented with 5% fetal calf serum (FCS; Thermo Fisher Scientific). Cells were incubated in a humidified incubator (Binder GmbH) at 37 °C containing 5% CO_2_. To prepare the stock solution, 10 mg mL^−1^ juglone was dissolved in dimethyl sulfoxide (DMSO; Roth). An appropriate volume of stock solution was mixed with culture medium to achieve the desired concentration, ensuring that the DMSO content did not exceed 0.1% in the final concentrations.

#### Cell viability assay

To investigate the cellular viability of HeLa cells in different concentrations of free juglone or JNPs, 3-(4,5-dimethylthiazol-2-yl)-2,5-diphenyltetrazolium bromide (MTT) assay was performed. Briefly, 10^5^ cells mL^−1^ suspension was inoculated into a 96-well plate (100 µL in each well) and incubated overnight at 37 °C with 5% CO_2_. Cells were then treated with free juglone at concentrations of 0, 5, 10, 15, 20, and 25 µM for 24 h. In parallel, equivalent doses of JNP were used, which were calibrated to release the same concentrations of juglone over a similar timeframe of 24-h. Cell viability was detected using the MTT reagent (Biofroxx GmbH). MTT solution was prepared by dissolving 5 mg mL^−1^ MTT in phosphate-buffered saline (PBS; Gibco) and filtered (0.22 µm, Whatman) for sterilization. After 24 h of treatment, 10 µL MTT solution was added to each well and incubated at 37 °C for 3 h. Afterward, the medium was discarded, and resulting formazan crystals were dissolved in 100 µL DMSO and incubated with gentle shaking at 37 °C for 15 min to ensure complete dissolution. Absorbance was measured at 560 nm immediately with a microplate reader (Biochrom ASYS, Expert 96). Control growth was considered to be 100% viable and relative viability was calculated as follows:

4 $${Cell\; viability}\left( \% \right)=\frac{{OD\; value\; of\; the\; treated\; cells}}{{OD\; value\; of\; control\; cells}}* 100$$

#### Western blotting

Given that approximately 50% cell viability was observed at 20 µM, and 25 µM induced a substantial increase in cell death, these concentrations were selected for further experiments to assess the impact of both moderate and high doses on protein expression. Western blot experiments were carried out to detect various levels of protein expression. Briefly, the supernatant of homogenized cell lysate was separated by centrifugation (1000 × g, for 10 min, at +4 °C), and its protein concentration was measured using Micro BCA Protein Assay Kit (Thermo Scientific). Each 30 µg of protein was separated via 14% sodium dodecyl sulfate polyacrylamide gel electrophoresis (SDS-PAGE; Bio-Rad) and transferred to a nitrocellulose membrane (Machenerey-Nagel). After primary (Cleaved caspase-3 (Asp175), Cell Signaling Technology, 9661; BCL-2, Santa Cruz Biotechnology, sc-7382; GAPDH, Abcam, ab181602) and HRP-conjugated secondary antibody (Dako) administration, Amersham ECL Western Blotting detection reagent (GE Healthcare) was used to visualize the membrane via ECL Imager (Intas). Densitometric quantification was performed using ImageJ software (National Institutes of Health).

#### Immunofluorescence staining

An anti-apoptotic protein, BCL-2 and an apoptotic protein, cleaved caspase-3 (cCas-3) expressions were detected by immunofluorescence staining as well. Therefore, HeLa cells were cultured in a 24-well plate, fixed with 4% paraformaldehyde (PFA) and blocked with 5% BSA in PBS. Primary antibodies were incubated overnight in a humidified dark chamber at +4 °C. After the washing steps, the Cy3-conjugated secondary antibody (1:250, Dianova) was incubated at room temperature for 1 h. DAPI (Sigma-Aldrich) was added to the secondary antibody in order to co-label nuclei. Fluorescence microscopy images were acquired via Axio Imager 2 (Zeiss) and were evaluated according to positive signal intensity using ImageJ software (National Institutes of Health) and normalized for DAPI.

#### TUNEL assay

TUNEL assay was conducted to detect DNA fragmentation, thus apoptosis. To identify apoptotic cells, TUNEL assay kit (BrDU-Red, ab66110, Abcam) has been used according to the manufacturer’s protocol. HeLa cells cultured on coverslips were treated with juglone and JNP when cell confluency reached80%. After 24 h of treatment, cells were fixed in 4% paraformaldehyde and washed with PBS, followed by incubation with proteinase K solution for 5 min, at room temperature. Afterward, the DNA labeling solution was added to the coverslips. Finally, an anti-BrdU (red signal) antibody was added and cell nuclei were counterstained with DAPI (4′,6-diamidino-2-phenylindole, blue signal, Sigma-Aldrich). Fluorescence images were acquired (Axio Imager 2, Zeiss). The apoptosis rate was calculated as the ratio of TUNEL-positive nuclei per total number of nuclei (DAPI; blue) per optic field. Image analysis was performed with ImageJ software (National Institutes of Health).

#### Wound healing assay

A wound-healing assay was conducted to see the effects of juglone and JNP on cellular growth and migration. Subsequently, a monolayer of HeLa cells were grown on coverslips (confluency reached 90%). The culture medium was thereafter replaced with the treatment medium. Next, a smooth line was scratched with a sterile 200 µL pipette tip and imaging was carried out immediately, and 24 h post treatment. Scratch surface area was evaluated via ImageJ (National Institutes of Health).

#### Statistical analysis

All data are expressed as means ± SD from at least three independent experiments. Differences between groups were analyzed by Student’s *t*-test. A significance level of *p* < 0.05 was considered statistically significant. Non-significant results were represented as “ns.”

## Results

### Nanoparticle Production and Characterization

**Fig. 1:**
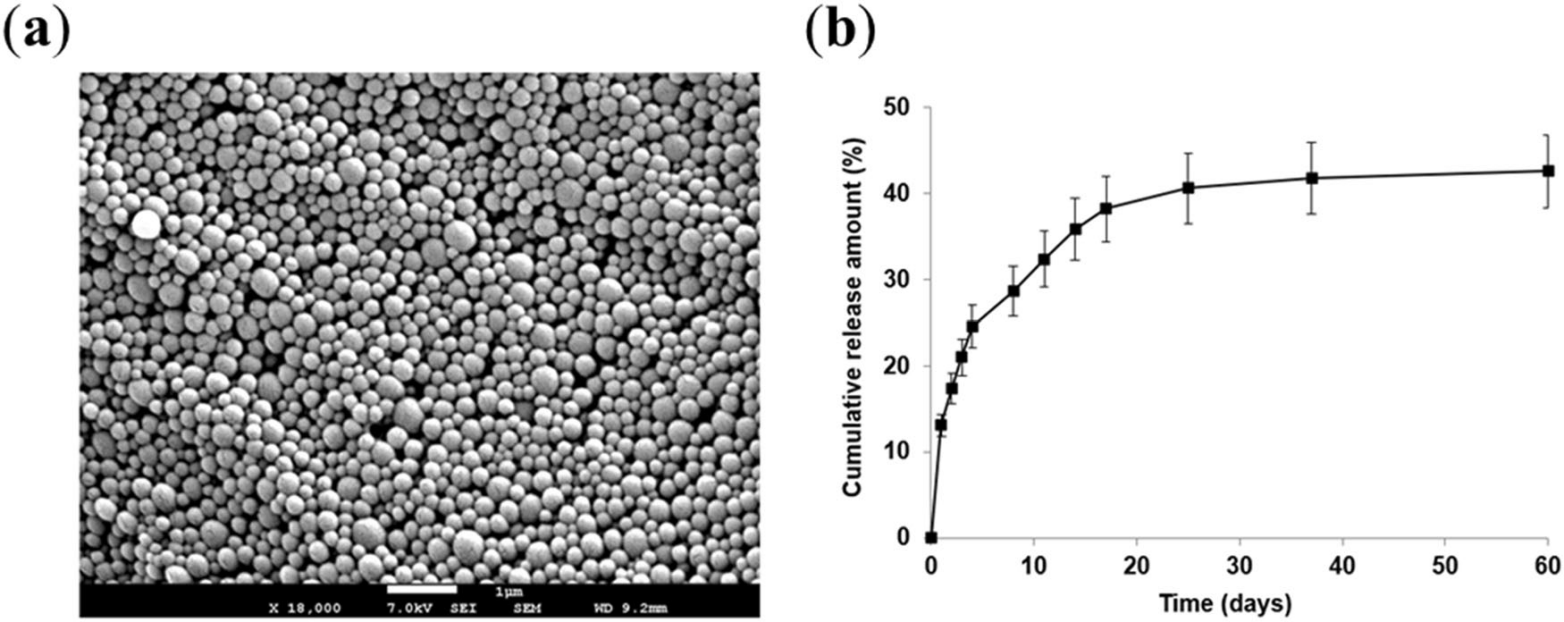
SEM image of juglone-loaded PLGA nanoparticles (**a**) and their time-dependent drug release profile over 60 days (**b**)

The encapsulation efficiency (EE), reaction yield (RY), and drug loading (DL) of the juglone nanoparticles were determined to be 90.12, 66.90, and 47.80%, respectively. The JNP had a mean diameter of 207.45 ± 1.67 nm with a polydispersity index (PDI) of 0.153 ± 0.02, indicating a uniform size distribution. SEM analysis confirmed these findings, revealing spherical nanoparticles with consistently distributed sizes around 200 nm (Fig. 1a). Zeta potential measurements showed a value of –24.12 ± 2.21 mV, suggesting that the nanoparticles are stable and exhibit no significant aggregation in suspension. The release profile of the nanoparticles, monitored over 60 days, is presented in Fig. 1b. An initial burst release was observed within the first hour, followed by a sustained and gradual release pattern, reaching 42.57% by the end of the 60-day period.

### Anticancer Activity of Juglone Nanoparticles

#### Cell viability

**Fig. 2:**
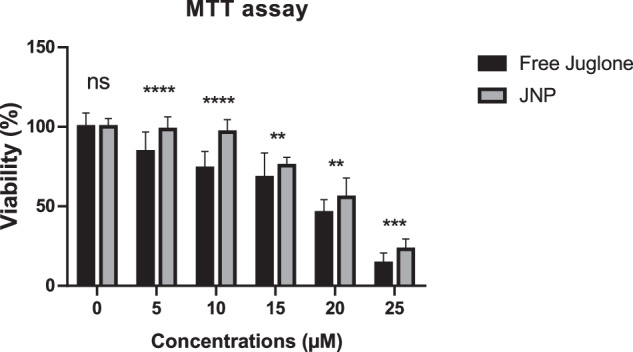
The cytotoxic effects of free juglone and juglone nanoparticles (JNP) on HeLa cells. Proliferation and growth inhibitory effect of free juglone and JNPs on HeLa cells were determined by MTT assay. The HeLa cells were treated with different concentration range from 0 to 25 μM of juglone and JNPs for 24 h. Data are presented as mean ± SD; **p < 0.01, ***p < 0.001, and ****p < 0.0001

The effects of free juglone and JNP on cell viability were evaluated with the MTT assay. The results demonstrated a dose-dependent decrease in cell viability for both free juglone and JNPs (Fig. 2). At the highest concentration of 25 µM, 14% of cells remained viable in the free juglone-treated group. In contrast, 23% of cells were viable in the JNPs-treated group. The half-maximal inhibitory concentration (IC_50_) values for free juglone and JNP were 17.07 µM and 20.64 µM, respectively (Supplementary Fig. 1) The similar trends in cell viability between free juglone and JNP further validated the accuracy of the release study and the corresponding calculations.

#### Western blotting

**Fig. 3:**
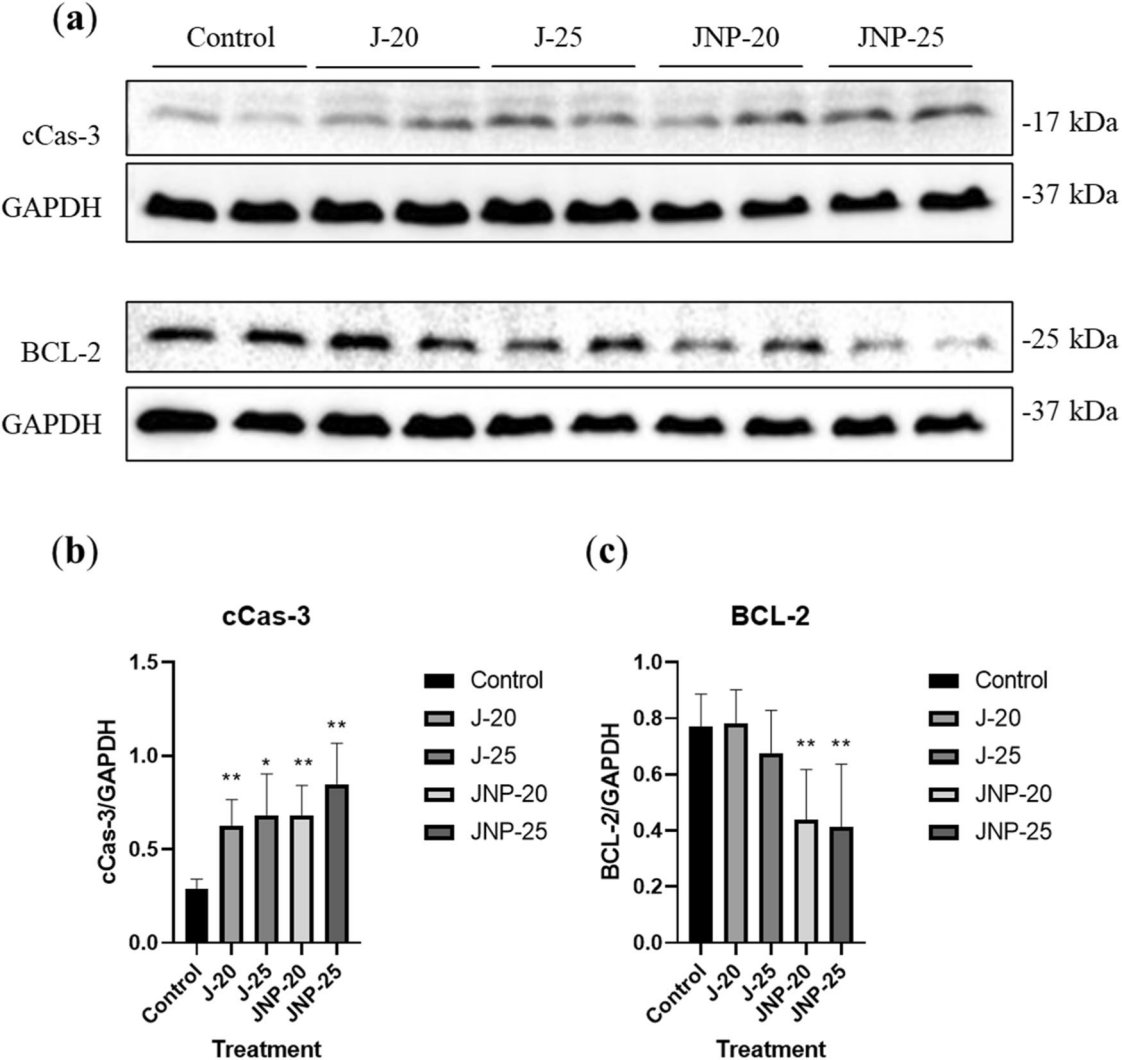
Effect of different concentrations (20 and 25 μM) of free juglone and juglone nanoparticles on expression of apoptosis-related proteins in HeLa cells. **a** Protein expression levels of cleaved caspase-3 (cCas-3) and BCL-2 were detected by western blotting. GAPDH was used as loading control. **b** Bar graph demonstrate the densitometric evaluation of the levels of cCas-3 from three independent experiments. **c** Bar graph demonstrate the densitometric evaluation of the levels of BCL-2, from three independent experiments. Data are the means ± SD; *p < 0.05, **p < 0.01. J-20: 20 μM free juglone, J-25: 25 μM free juglone, JNP-20: 20 μM juglone nanoparticles and JNP-25: 25 μM juglone nanoparticles

The expression of apoptosis-related proteins was examined via western blot analysis. The results (Fig. 3a, b, Supplementary Fig. 2a, b) showed that both free juglone and JNP significantly increased the expression of cCas-3, a well-known marker of apoptosis. The highest expression of cCas-3 was observed in the cells treated with 25 µM juglone NPs. In contrast, treatment with JNP at 20 and 25 µM concentrations significantly reduced BCL-2 levels, while free juglone treatment did not result in a significant decrease in BCL-2 expression (Fig. 3a–c, Supplementary Fig. 2c, d).

#### Immunofluorescence staining

**Fig. 4:**
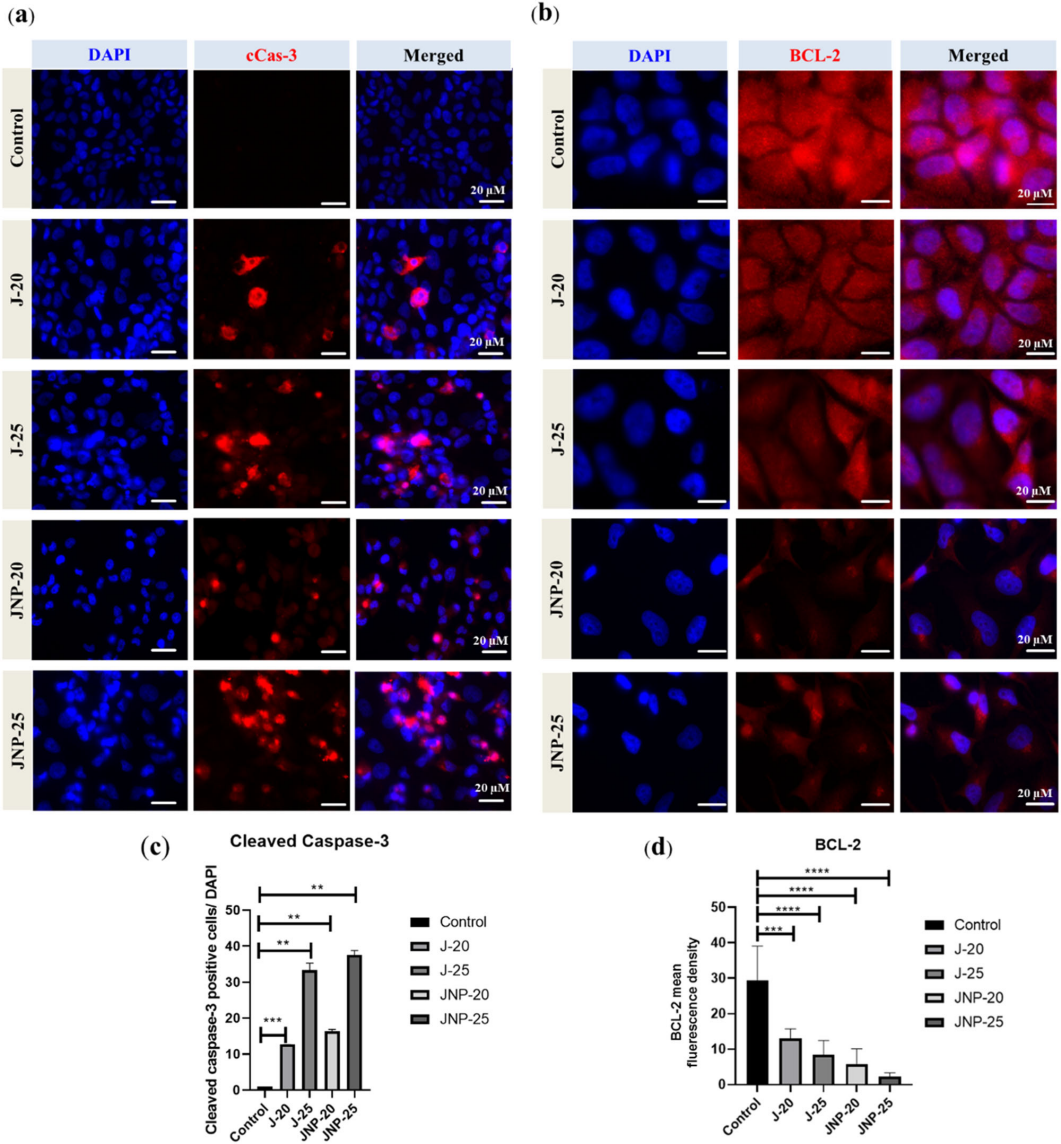
Effect of different concentrations (20 and 25 μM) of free juglone and juglone nanoparticles on expression of apoptosis-related proteins in HeLa cells. **a** Representative confocal microscopy images of cCas-3 and BCL-2 (red signal; **a** and **b**) in HeLA cells treated with free juglone and juglone nanoparticles. Nuclei were counterstained with DAPI (blue signal). **c** The bar graph shows numerical evaluation of cCas-3-positive cells normalized to total cell numbers in the analyzed regions. **d** The bar graph shows mean fluorescence value of BCL-2. N = three independent experiments. Data are the means ± SD; **p < 0.01, ***p < 0.001, and ****p < 0.0001. J-20: 20 μM free juglone, J-25: 25 μM free juglone, JNP-20: 20 μM juglone nanoparticles and JNP-25: 25 μM juglone nanoparticles

Immunofluorescence staining (Fig. 4a–c) revealed a clear, dose-dependent increase in the expression of cCas-3 for both free juglone and JNP. cCas-3 is known to translocate from the cytoplasm to the nucleus during apoptosis [34], and to confirm this translocation, a nuclear-specific fluorescent dye (DAPI) was used to ensure accurate signal localization. Furthermore, BCL-2 expression was found to be decreased in a dose-dependent manner as the juglone concentration increased (Fig. 4b–d). A reduction in BCL-2 expression is a well-known indicator of apoptosis [35], making the apoptotic effect of juglone more evident in the immunofluorescence analysis compared to the western blot results. Notably, the reduction in BCL-2 expression was more pronounced with JNP than with free juglone, suggesting that encapsulation in PLGA enhances the therapeutic activity of juglone.

#### TUNEL assay

**Fig. 5:**
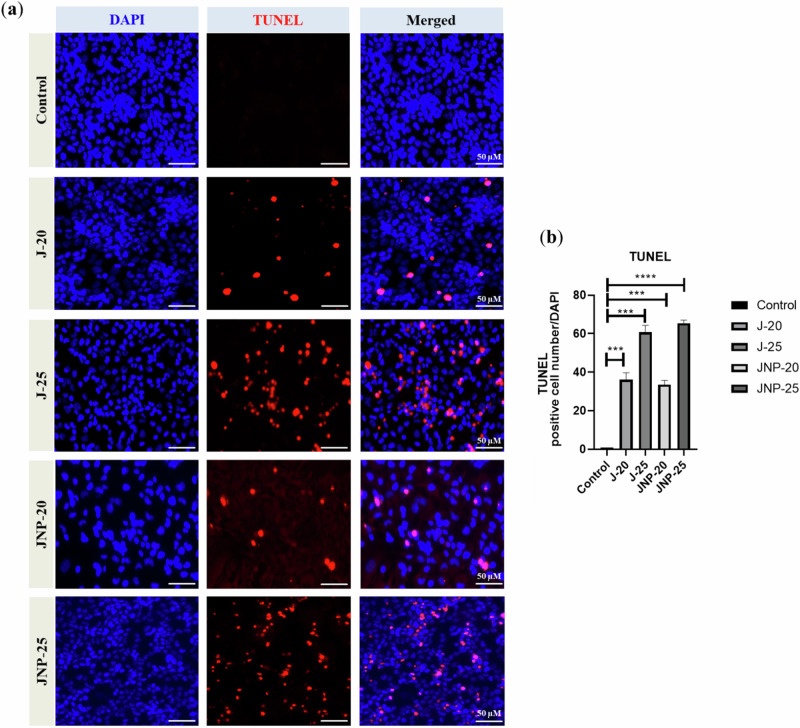
Free juglone and juglone nanoparticles-mediated nuclear DNA fragmentation of HeLa cells and cell death detected by the TUNEL assay. **a** TUNEL-positive (BrdU, red) apoptotic cells were examined under fluorescence microscope. Nuclei were counterstained with DAPI (blue signal). **b** The bar graph shows the quantitative analysis of TUNEL-positive HeLa cells. N = three independent experiments. Data are the means ± SD; ***p < 0.001, and ****p < 0.0001. J-20: 20 μM free juglone, J-25: 25 μM free juglone, JNP-20: 20 μM juglone nanoparticles and JNP-25: 25 μM juglone nanoparticles

In the TUNEL assay, both DAPI and anti-BrdU (Bromodeoxyuridine/5-bromo-2’-deoxyuridine) stainings were performed. BrdU incorporation into DNA strand breaks serves as a marker for apoptosis. The results demonstrated that increasing doses of juglone induced apoptosis in HeLa cells (Fig. 5a, b). There was no significant difference between free juglone and JNP in terms of apoptosis induction.

#### Wound healing

**Fig. 6:**
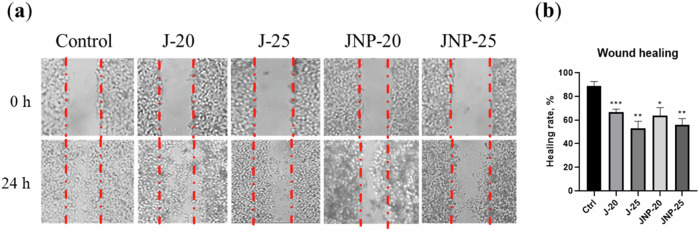
The effects of free juglone and juglone nanoparticles on HeLa cell migration and invasion. **a** Cell migration was determined using scratch (wound-healing) assay. **b** The bar graphs show the quantification of the wound healing rate. N = three independent experiments. Data are the means ± SD; *p < 0.05, **p < 0.01. and ***p < 0.001. J-20: 20 μM free juglone, J-25: 25 μM free juglone, JNP-20: 20 μM juglone nanoparticles and JNP-25: 25 μM juglone nanoparticles

The light microscopy images showed that juglone and JNP successfully inhibited cell migration and proliferation of HeLa cells (Fig. 6a). Healing rates were calculated in comparison with the control and the effects of free juglone and JNP were found to be similar, with healing rates of approximately 67% for 20 µM free juglone, 53% for 25 µM free juglone, 64% for 20 µM JNP and 56% for 25 µM JNP (Fig. 6b).

## Discussion

Juglone, a naturally occurring compound with a wide range of biological activities, has demonstrated significant anticancer potential across various cancer types. Despite its therapeutic potential, its clinical application is still limited by inherent challenges, including high toxicity and poor aqueous solubility, which adversely affect its efficacy and therapeutic index [36, 37]. To address these challenges, the development of nanoformulations has emerged as a promising strategy, offering improved therapeutic activity and reduced toxicity. In this study, we aimed to encapsulate juglone within PLGA nanoparticles, a biocompatible and biodegradable polymer, to enhance its solubility and mitigate its cytotoxic effects, thereby advancing its potential for clinical applications.

Our study reveals that the IC_50_ value of free juglone (17.07 µM) was slightly lower than that of JNPs (20.64 µM), as determined through MTT assays performed on HeLa cells. This difference may reflect the controlled and sustained release profile of the nanoparticle formulation These findings are consistent with previous studies, which reported that juglone significantly decreased HeLa cell proliferation, with a comparable IC_50_ value [8, 9, 38]. In alignment with our findings, Yue et al. reported that JNPs exhibit slightly lower IC_50_ values compared to free juglone in melanoma cells. Despite this, both free juglone and JNPs demonstrated comparable anticancer efficacy, suggesting that the sustained release properties of JNPs may contribute to their therapeutic potential while reducing systemic toxicity. These observations further underscore the advantages of nanoparticle-based delivery systems in enhancing drug performance and safety profiles for clinical applications [27]. The cell viability results for juglone and JNPs followed a similar trend, supporting the accuracy of our release study and demonstrating that the nanoparticle system achieves comparable effects at lower doses, as intended. Consistent observations were made in a previous study on juglone-BSA nanoparticles and free juglone using HT29 and A431 cell lines, where juglone-BSA nanoparticles showed significantly reduced cytotoxicity compared to free juglone at lower concentrations. However, the difference was less pronounced at higher concentrations [28]. Erisen et al. demonstrated that JNPs exhibit significantly reduced cytotoxicity to normal cells compared to free juglone, with an IC_50_ of 270 µmol/L for JNPs versus 60 µmol/L for free juglone in L929 fibroblast cells. This reduction in toxicity is attributed to the controlled release properties of the nanoparticles, which minimize the immediate effects of juglone. Incorporating such nanoparticulate systems may represent a promising strategy to mitigate the adverse effects commonly associated with juglone treatments [30].

Apoptosis, a tightly regulated process involved in both physiological and pathological conditions, is controlled by several key factors, including the BCL family of proteins. Among these, BCL-2 is an anti-apoptotic protein whose reduced expression is a hallmark of apoptosis. Another critical indicator of apoptosis is the activation of caspase proteins, with caspase-3 playing a central role in both intrinsic and extrinsic apoptosis pathways [39]. In our study, western blot analysis revealed that both free juglone and JNP treatments increased the expression of cCas-3; however, only JNP treatment effectively suppressed BCL-2 expression. This finding was further corroborated by immunofluorescence staining, which demonstrated that the PLGA nanoparticle system enhances the apoptotic activity of juglone. Additionally, a recent study investigating the molecular mechanisms of juglone-induced apoptosis in cervical cancer HeLa cells supports our findings. The study showed that juglone significantly reduced cell proliferation and upregulated apoptotic markers such as Bax, CytC, Fas, FasL, and caspase-3 via activation of the JNK/c-Jun pathway. Importantly, the use of a JNK inhibitor (SP600125) diminished the expression of these apoptotic proteins, confirming the pivotal role of the JNK pathway in juglone-mediated apoptosis [8]. These findings align with and complement our results, further highlighting the potent apoptosis-inducing capability of juglone. Previous studies have shown that juglone treatment reduces BCL-2 protein levels while increasing active caspase-3 levels in ovarian cancer SKOV3 and human breast cancer MCF-7 cells [12, 40]. The anticancer effect of the juglone molecule itself is believed to originate from the induction of apoptosis by increasing mitochondrial depolarization and ROS level, the alteration of the expression profile of apoptosis-related genes (elevated ratio of Bax/BCL‑2, activation of Cas‑3 and Cas‑9) [3]. In our study, the JNP system demonstrated a more pronounced pro-apoptotic effect compared to free juglone, as evidenced by greater cCas-3 activation and a more significant reduction in BCL-2 levels. These findings suggest that the nanoparticle formulation enhances the apoptosis-inducing potential of juglone.

The TUNEL assay, used to detect DNA fragmentation, revealed increased apoptosis in HeLa cells following treatment with both free juglone and JNPs. Notably, the highest apoptotic effect was observed at the maximum dose (25 µM) of JNPs, demonstrating the enhanced efficacy of the nanoparticle formulation. These results are consistent with the findings of Nassir et al., who reported a similar increase in apoptosis with resveratrol-loaded nanoparticles [41].

Animal experimental results provide compelling evidence for the therapeutic potential of JNPs. Yue et al. [27] conducted a study in a melanoma xenograft mouse model, where JNP significantly inhibited tumor growth compared to free juglone. Importantly, the nanoparticle formulation demonstrated markedly reduced systemic toxicity, as shown by minimal weight loss and stable histopathological profiles of major organs, including the heart, liver, spleen, lungs, and kidneys. In contrast, free juglone treatment resulted in significant weight loss and elevated liver enzyme levels (ALT and AST), indicative of hepatotoxicity. These findings underscore the advantages of nanoformulation in enhancing drug delivery and minimizing off-target effects. Furthermore, biodistribution studies in the same model confirmed the tumor-targeting properties of JNP, attributed to the enhanced permeability and retention effect [42], which facilitates prolonged retention of the nanoparticles at tumor sites. This observation aligns with the improvement in therapeutic efficacy and safety, reinforcing the clinical potential of JNP [27].

A wound healing assay was conducted to evaluate the effect of JNP on cell migration, a crucial step in metastasis. The findings revealed that both free juglone and JNP effectively inhibited cell migration. These results are consistent with the study by Yue et al., which demonstrated that juglone nanoparticles exhibit significant anti-migration effects on melanoma cells [27].

While our study demonstrates the potential of JNPs in cultured HeLa cells, several limitations need to be addressed. A key limitation of our NP system is the lack of tumor-specific targeting. The current formulation is based on passive targeting which may lead to non-specific, off-target effects. Nair et al. reported that non-targeted therapeutic strategies result in a very low proportion of the administered drug dose reaching the tumor site [43]. Additionally, our study is limited to in vitro experiments, and the lack of in vivo studies prevents a comprehensive evaluation of the efficacy, biodistribution, and safety of JNPs.

All these results demonstrate that encapsulating juglone within PLGA nanoparticles improved its solubility, enabled controlled drug release, reduced toxicity, and enhanced its apoptotic effects. These improvements are critical for achieving better therapeutic outcomes. Importantly, nanoformulations also offer the potential to selectively target tumor cells, minimizing damage to healthy tissues.

## Conclusion

This study underscores the potential of nanotechnology to optimize the therapeutic profile of natural compounds like juglone, a bioactive metabolite with significant anticancer properties but limited clinical applicability due to its hydrophobicity and toxicity. By encapsulating juglone in PLGA nanoparticles, we successfully enhanced its water solubility and achieved controlled release, leading to comparable antiproliferative effects with free juglone at lower doses. Importantly, the nanoparticle formulation amplified the apoptotic response, as evidenced by enhanced caspase-3 activation and reduced BCL-2 expression. These findings highlight the ability of PLGA-based nanocarriers to mitigate the limitations of juglone, offering a safer and more effective platform for its delivery. However, to further validate the clinical applicability of this system, future studies will focus on conducting in vivo experiments in animal models to evaluate the safety, biodistribution, and therapeutic performance of the nanoparticles under physiological conditions. Additionally, we aim to enhance the specificity and efficacy of the delivery system by modifying the nanoparticles with targeted receptors, enabling receptor-mediated delivery of juglone to cancer cells. These advancements could pave the way for the development of more precise and effective nanoparticle-based therapies for cancer treatment.

## Supplementary information

Supplementary Figures

## Data Availability

No datasets were generated or analysed during the current study.
