# Supplementary material for: Juglone Encapsulation in PLGA Nanoparticles Improves Solubility and Enhances Apoptosis in HeLa Cells
Source: Cell Biochem Biophys. 2025 Feb 14;83(3):3081–92. doi: 10.1007/s12013-025-01691-9 (PMC12414070; doi:10.1007/s12013-025-01691-9)
Supplement: Supplementary file 1 — Supplementary Figures [file 12013_2025_1691_MOESM1_ESM.docx]

**Supplementary Figures**

**
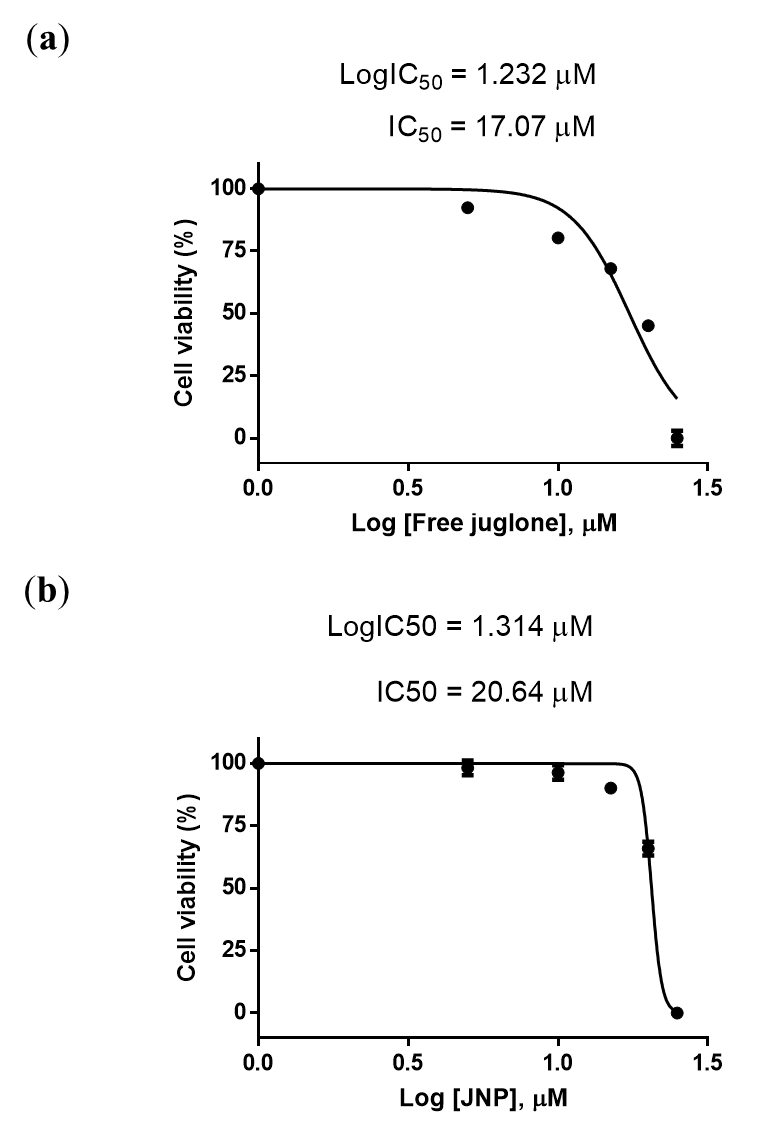
**

**Supplementary Fig. 1.** IC_50_ graphs. The figures show the 50% inhibitory concentrations (IC_50_) of the free juglone (a) and JNP (b) in HeLa cell lines at 24 h.


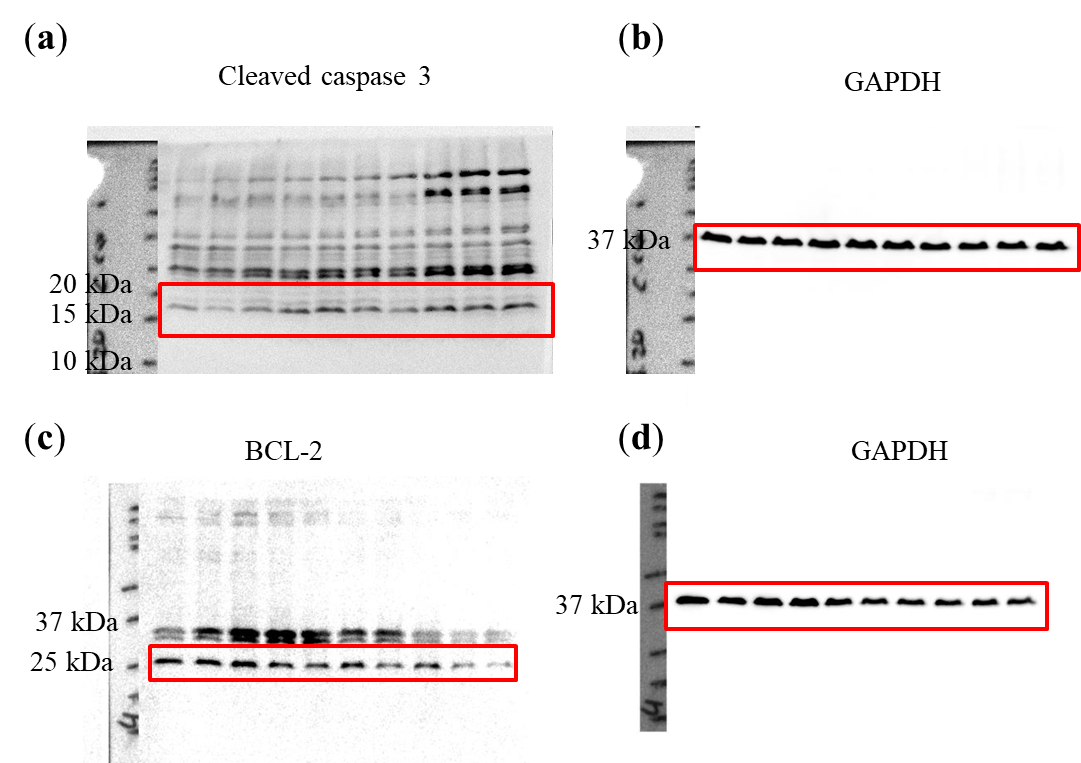


**Supplementary Fig. 2.** Uncropped western blot images. (a-d) Uncropped Western Blot images displayed in Fig. 3a, as well as the approximate extent of the cropped region (red box).
